# Telomere length is a prognostic biomarker in elderly advanced ovarian cancer patients: a multicenter GINECO study

**DOI:** 10.18632/aging.100840

**Published:** 2015-12-03

**Authors:** Claire Falandry, Béatrice Horard, Amandine Bruyas, Eric Legouffe, Jacques Cretin, Jérôme Meunier, Jérôme Alexandre, Valérie Delecroix, Michel Fabbro, Marie-Noëlle Certain, Raymonde Maraval-Gaget, Eric Pujade-Lauraine, Eric Gilson, Gilles Freyer

**Affiliations:** ^1^ Geriatrics and Oncology Unit, HCL Cancer Institute, LBMC, CarMEN Laboratory, Lyon 1 University, Lyon, France; ^2^ LBMC, ENS/Lyon, Lyon 1 University,CGphiMC Lyon 1 University, Lyon, France; ^3^ Oncology Unit, Lyon Sud University Hospital, Lyon University, Pierre-Bénite, France; ^4^ Clinique Valdegour, Department of Medical Oncology, Nîmes, France; ^5^ Clinique Bonnefon, Oncology and Radiotherapy Department, Alès, France; ^6^ Centre Hospitalier Régional d'Orléans, Department of Medical Oncology, Orléans, France; ^7^ Paris Descartes University, AP-HP, Hôpitaux Universitaires Paris Centre, Site Hôtel Dieu, Paris, France; ^8^ Clinique Mutualiste de l'Estuaire, Cité Sanitaire, Department of Medical Oncology, Saint-Nazaire, France; ^9^ Institut du Cancer Montpellier, Medical Oncology, Montpellier, France; ^10^ Centre Hospitalier d'Auxerre, Department of Medical Oncology, Auxerre, France; ^11^ Lyon Sud University Hospital, Pierre-Bénite, France; ^12^ LBMC, Lyon 1 University, IRCAN, CNRS UMR 7284, INSERM U1081, Nice Sophia-Antipolis University; CHU of Nice, Nice, France; ^13^ HCL Cancer Institute, Department of Medical Oncology, Lyon 1 University, Lyon, France

**Keywords:** telomere, ovarian cancer, elderly, geriatric, prognostic factor

## Abstract

**Purpose:**

Age induces a progressive decline in functional reserve and impacts cancer treatments. Telomere attrition leads to tissue senescence. We tested the hypothesis that telomere length (TL) could predict patient vulnerability and outcome with cancer treatment.

**Patients and methods:**

An ancillary study in the Elderly Women GINECO Trial 3 was performed to evaluate the impact of geriatric covariates on survival in elderly advanced ovarian cancer patients receiving six cycles of carboplatin. TL was estimated from peripheral blood at inclusion using standard procedures.

**Results:**

TL (in base pairs) was estimated for 109/111 patients (median 6.1 kb; range [4.5-8.3 kb]). With a cut-off of 5.77 kb, TL discriminated two patient groups, long telomere (LT) and short telomeres (ST), with significantly different treatment completion rates of 0.80 (95%CI [0.71-0.89]) and 0.59 (95%CI [0.41-0.76]), respectively (odds ratio [OR]=2.8, p=0.02). ST patients were at higher risk of serious adverse events (SAE, OR=2.7; p=0.02) and had more unplanned hospital admissions (OR=2.1; p=0.08). After adjustment on FIGO stage, TL shorter than 6 kb was a risk factor of premature death (HR=1.57; p=0.06).

**Conclusion:**

This exploratory study identifies TL as predictive factor of decreased treatment completion, SAE risk, unplanned hospital admissions and OS after adjustment on FIGO stage.

## INTRODUCTION

Aging is associated with a progressive decline in the functional reserve of multiple organ systems [1]. Given that the process of aging is heterogeneous, this decline should ideally be assessed individually and care of an elderly person adapted accordingly rather than solely on the basis of chronological age. Such assessments are currently entirely clinical, based on a geriatric evaluation.

During normal ageing, the gradual loss of telomeric DNA in dividing somatic cells contributes to replicative senescence [2]. Importantly, this telomere length dynamics plays an important signaling role in determining cell fate during aging and cancer [3]. There is growing evidence linking pathologic aging to telomere shortening in prospective studies recruiting elderly patients, although there is some controversy associated with this research. Patients with shorter telomeres tend to develop more functional disabilities [4], have increased cognitive loss [5], higher cardiovascular morbidity [6], more degenerative diseases [7] and higher mortality [8].

In an oncologic context, the impact of aging on a patient's survival is challenged by the nature of the tumor itself, which in turn means a differential impact of geriatric covariates on overall survival (OS). In 1997, the French National Group of Investigators for the Study of Ovarian and Breast Cancer (GINECO) established a research program focused on the treatment of ovarian cancer in elderly women. The feasibility of carboplatin-cyclophosphamide and standard carboplatin AUC5-paclitaxel protocols in patients over 70 years of age was demonstrated in two studies [9,10], with treatment completion rates of 76% and 68% respectively [10]. A multivariate analysis performed in a non-randomized retrospective review of these trials reported a significant negative impact of various geriatric covariates on survival [10]. A prospective trial, the Elderly Woman GINECO Trial 3, was thus initiated to evaluate the impact of geriatric covariates on survival in elderly patients with advanced ovarian cancer treated with six cycles of carboplatin AUC5. A geriatric vulnerability score (GVS) was developed which segregates patients into two groups with significantly different outcomes in terms of treatment completion rates and risk of treatment toxicities (serious and severe adverse events, unplanned hospital admissions, [11]).

An ancillary study was envisaged in the original design of the GINECO Trial 3 with a working hypothesis that telomere biology influences patients' future outcomes and may correlate with a clinical geriatric assessment. The results reported here pinpoint an association between short TL, treatment tolerance and completion in ovarian cancer patients.

## RESULTS

### An overall decrease in telomere length with age in the patient cohort used in this study

Duplicate telomere length (TL) distribution measurements were performed on blood samples from 109 of the 111 patients included between August 2007 and January 2010. Patient characteristics and outcome of the geriatric assessment are shown in Table 1. Median follow-up was 16.4 months (range 0.2-49.6).

**Table 1:** Patient and disease characteristics and geriatric assessment

	N of patients (%)
Median age in years (range)	78 (70-93)
≥80 years	44 (40.3)
Performance status (ECOG) ≥2	51 (46.8)
Tumor assessment	
FIGO stage IV	38 (34.9)
Complete primary cytoreduction	18 (16.5)
Geriatric assessment	
≥3 comorbidities	26 (23.9)
N comedications	
1-3	32 (29.4)
4-6	44 (40.4)
≥7	30 (27.5)
Functional assessment	
ADL score <6	60 (55.0)
IADL score <25	76 (69.7)
Nutritional assessment	
Albuminemia <35 g/L	64 (58.8)
BMI <21 kg/m.	24 (22.0)
Lymphocyte count <1 G/L	27 (24.8)
Psychocognitive assessment	
MMS score <25	32 (29.4)
HADS score >14	40 (36.7)
GDS score >10	39 (35.6)

ADL: Activities of Daily Living; IADL: Instrumental Activities of Daily Living; BMI: Body Mass Index; ECOG: Eastern Cooperative Oncology Group; GDS: Geriatric Depression Scale; HADS: Hospital Anxiety and Depression Scale; MMS: Mini-Mental Scale

**Figure 1:**
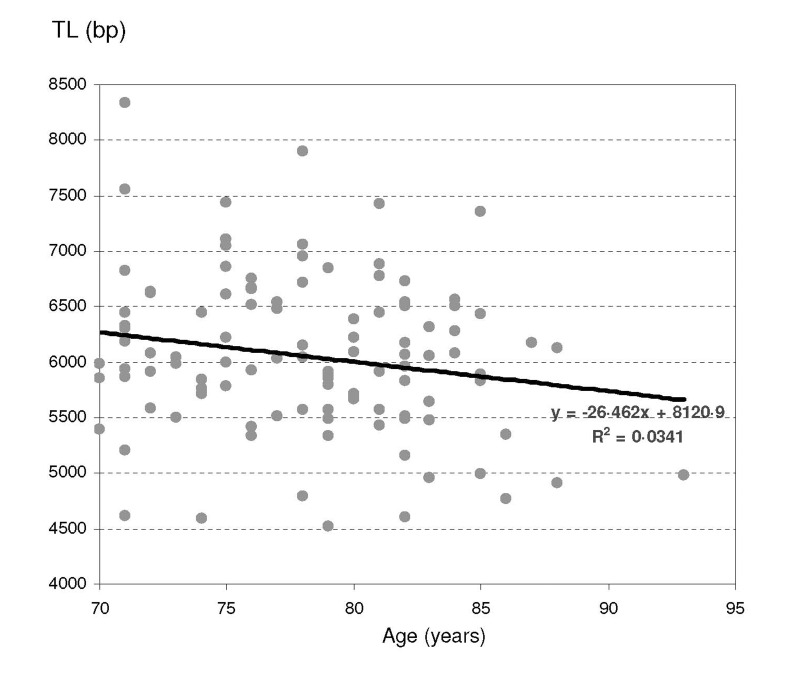
Telomere length repartition according to age

TL ranged from 4.52 to 8.33 kilobases (kb) with a mean of 6.05 kb (SD 0.71 kb). A weak inverted linear correlation with age was demonstrated, with every 1-year increase in age associated with a 26-base pair decrease in mean TL, with a R. ratio of 0.0341 (Figure 1).

### Longer TL in patients that completed their treatment and exhibited a better tolerance

Since treatment completion is considered a meaningful short-term marker of patient outcome, we analyzed for a possible correlation between TL distribution and the fact that patients completed their treatment. In order to analyze variations in the non-gaussian distributions of human TL, we segregated the patients according to TL quartiles (Table 2). Patients with telomeres longer than 6.54 kb had a 52% higher chance of treatment completion than those with telomeres shorter than 5.77 kb (95%CI: 1.00-2.32, *P*=0.04). Thus, we can identify two groups of patients with different treatment completion rates according to their TL distribution: 80% (95%CI: 71% to 89%) in the group with telomeres longer than 5.77 kb (LT group) versus 59% (95%CI: 41% to 76%) in the group with shorter telomeres (ST group, *P*=0.02).

**Table 2:** Association between telomere length parameters and patient outcomes

	Telomere length	Odds ratio for treatment completion (95%CI)	*P-value*	Hazard ratio for death (95%CI) (*adjusted for FIGO stage)*	*P-value*
TL mean	6.05 kb	0.56 (0.22-1.27)	*0.15*	1.42 (0.88-2.29)	*0.15*
TL median	6.00 kb	0.50 (0.21-1.2)	*0.11*	1.57 (0.98-2.51)	*0.06*
TL quartiles		**1.50 (1.01-2.23)**	***0.04***	**0.82 (1.67-1.01)**	***0.07***
	**<5.77 kb**	**0.36 (0.15-0.87)**	***0.02***	1.49 (0.91-2.44)	*0.12*
	5.77-6.06 kb	1.60 (0.54-4.74)	*0.38*	1.02 (0.59-1.77)	*0.94*
	6.06-6.54 kb	1.28 (0.46-3.58)	*0.64*	0.97 (0.55-1.72)	*0.91*
	>6.54 kb	2.08 (0.65-6.67)	*0.19*	0.62 (0.34-1.14)	*0.12*

Using the same cut-off of 5.77 kb, TL segregates the same two groups (i.e., ST vs LT) as having different outcomes in terms of tolerance. Serious adverse events were significantly more frequent in the ST group, with an odds ratio of 2.7 (*P*=0.02). Unplanned hospital admissions and grade 3-4 non-hematological toxicity also tended to be more frequent (Table 3). No significant difference between TL groups could be identified in terms of hematological toxicity, however blood cell counts were only routinely evaluated 1 day prior to chemotherapy.

**Table 3:** TL repartition of vulnerability criteria and clinical end points between

	Observed risk:short/long telomere group	95% CI	P-value
Treatment completion	0.36	0.15-0.87	0.020
Serious Adverse Events	2.69	1.17-6.19	0.019
Unplanned hospital admissions	2.14	0.92-4.95	0.076
Grade ≥ 3 non-hematological toxicity	2.04	0.88-4.71	0.095
Grade ≥ 3 hematological toxicity	1.32	0.58-3.00	0.51
**Geriatric vulnerability parameters**:			
ADL score < 6	1.78	0.77-4.12	0.17
IADL score < 25	1.31	0.53-3.22	0.56
HADS score >14	1.89	0.82-4.33	0.13
Albuminemia <35 g/L	1.27	0.55-2.90	0.57
Lymphocytes <1 × 10^9^/L	1.76	0.71-4.36	0.22
**Geriatric vulnerability score**			
GVS ≥3	2.06	0.90-4.70	0.08

### Correlation between TL distribution and overall survival

**Figure 2:**
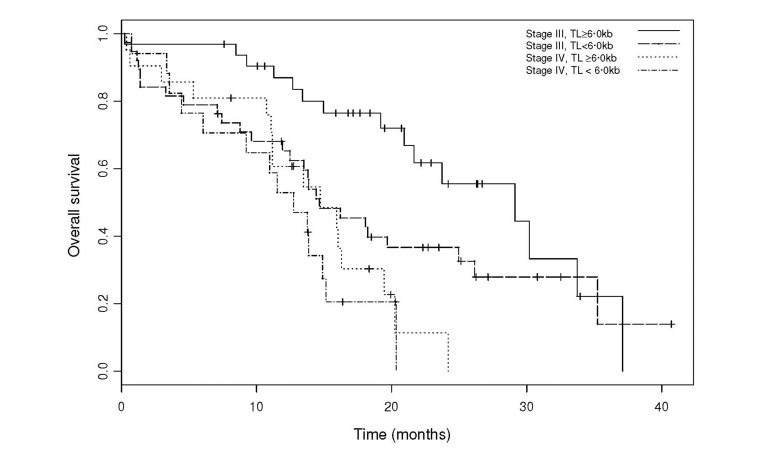
Overall survival by TL groups, adjusted for FIGO stage

A survival analysis using Cox proportional hazards was conducted in order to evaluate the impact of telomere length on survival (Figure 2). Reasons for death were the most frequently related to cancer progression (105 patients), 2 patients died from treatment toxicity (septic shock), 4 from other reasons (colic perforation: 1; pulmonary embolism: 1, suspicion of pulmonary embolism: 1, major depression and denutrition: 1). After adjustment on FIGO stage (IV versus III), TL less than 6.00 kb was identified as a risk factor for premature death, with an HR of 1.57 (95%CI: 0.98 to 2.51, *P=*0.06). This pejorative trait staid robust in different models including FIGO stage and age [HR = 1.58 (95%CI: 0.99 to 2.53, P=0.06)], FIGO stage and GVS . 3 [HR = 1.56 (95%CI: 0.97 to 2.49, P=0.07)] and FIGO stage, GVS . 3 and age [HR = 1.57 (95%CI: 0.98 to 2.53, P=0.06)] (Supplementary Table 1).

### A tendency towards correlation between TL and geriatric vulnerability parameters

We then tested the correlation between TL groups and geriatric vulnerability groups using the Geriatric Vulnerability Score [11]. Patients displaying at least three geriatric vulnerability parameters had a 2.94-fold higher risk of mortality in a univariate analysis (95%CI 1.79-4.84, *P* <0.0001) and this was 2.89-fold in a multivariate analysis after adjustment for FIGO stage (95%CI 1.74-4.78, *P*<0.0001). Despite the absence of a significant correlation with patients' characteristics except age (Supplementary Table 2) and any of the individual geriatric vulnerability parameters, TL groups and geriatric vulnerability groups showed a tendency towards correlation (*P*=0.08, Table 3).

## DISCUSSION

This analysis was planned as an ancillary study in the prospective multicentric Elderly Woman GINECO Trial 3. The working biological hypothesis - identifying TL as a putative prognostic biomarker in elderly cancer patients - was based on the pooled results of two prior studies, Elderly Woman GINECO Trials 1 and 2. In a retrospective multivariate analysis of overall survival (OS), several factors - FIGO stage IV, use of paclitaxel, age, emotional disorders and lymphopenia - were significantly associated with an increased risk of premature death ([10] and unpublished data). Moreover, a significant correlation was also shown between emotional disorders and lymphopenia. TL shortening, previously shown to correlate with age [13,14], lifestyle stress [15] and survival [10], is considered to be both an actor and a witness of the pathologic aging process. The myeloid skewing of hematological progenitors that accompanies aging [16] and telomere dysfunction [17,18] could explain why there is an increasing amount of clinical data proposing lymphopenia as a marker of pathologic aging [19].

In previous epidemiological cohorts, a correlation was observed between TL and lifespan [8,20,21], aging associated diseases [7], and OS in healthy subjects [22]. These findings are of limited clinical use since associations only appear when large cohorts are investigated and the predictive value on an individual basis is poor. TL in blood leucocytes has also gained considerable interest as a potential biomarker of cancer risk, and direct measurement of TL and telomerase activity in tumors are considered to be cancer prognosis markers [23, 24].

To our knowledge, this study is the first to investigate the impact of TL on treatment feasibility as an individual risk factor. In spite of a relatively small patient sample size (111 patients), we were able to control several putative biased errors typically associated with TL measurement in clinical trials [25–27]. All patients were female, post-menopausal, almost exclusively Caucasian and fell into a narrow age bracket (70 to 93 years). According our biologic working model, the impact of TL shortening was expected to be challenged by the competition between tumor-related and host-related covariates on patients' outcomes. Due to the context of the trial, the study did not include any external reference, as for example TL samples from non-cancer elderly patients, that would have evaluated the impact of the tumor itself on TL.

Despite these constraints, in this particular oncologic context of ovarian cancer, our results reveal a clear correlation between TL and patient immediate outcomes. Indeed, TL distribution identified a subgroup of elderly patients with short telomeres who have a lower probability of completing treatment and a higher risk of severe adverse events and unplanned hospitalization This subgroup partially overlaps with patients identified as vulnerable according to the GVS [28]. However, the translation to clinical practice of these results might be difficult for the following reasons. Firstly and as usually for TL analyses, the cut-off between short and long telomeres were made *a posteriori,* being highly dependent on the technical conditions and the population studied. Moreover, different cut-offs separated the immediate outcomes (treatment completion, severe adverse events, unplanned hospital admissions) and the risk of premature death. Secondly, TL remained less discriminating than the GVS, based on simple clinical tests and routine bioassays, for immediate outcomes and survival. Finally, TL was estimated using the gold standard technique, namely the mean length terminal restriction fragments. Even if this technique is highly feasible, it is time-consuming and difficult to implement in routine analysis. In this respect, many of large epidemiological cohort analyses have preferred an alternative method of TL estimation, namely quantitative PCR [29,30]. However, this alternative technique suffers from a number of technical disadvantages, notably a high coefficient of variation and a lack of good reference standards, making it difficult to evaluate absolute TL [31]. Studies in mouse models have revealed that the number of dysfunctional telomeres is a more accurate factor than mean telomere length in the evolution of tissue pathology during aging [32]. Thus, evaluation of blood markers of dysfunctional telomeres [33] appear to be a promising method for telomere biology analysis in clinical trials [34, 35].

In conclusion, our study demonstrates a correlation between telomere length and patient outcomes in an oncogeriatric context [34]. This finding opens the way for future work aimed at identifying telomere biomarkers which can be implemented in routine practice for outcome prediction as well as on the evaluation of the impact of cancer treatment - mainly chemotherapy - on biomarkers of aging.

## MATERIALS AND METHODS

### Study design

The Elderly Woman GINECO Trial 3 was an open-label phase II multicentric trial approved by the Independent Ethics Committee of Lyon University Hospital (EUDRACT No. 2006-005504-13). The study design, population and assessments have been described elsewhere [1]. Written informed consent was obtained from each patient and included authorization for collection of a blood sample for TL measurement. Patients were treated with up to six cycles of carboplatin AUC5 (5 mg/mL/min for 30 min every 3 weeks). In this ancillary study, TL at inclusion was evaluated, along with the impact of TL on patient outcome and the correlation between TL and geriatric covariates.

###  Patient population

Eligible patients were ≥70 years old, with a life expectancy ≥3 months, and histologically or cytologically proven epithelial FIGO stage III-IV ovarian cancer. Cytology consistent with ovarian cancer was considered sufficient if associated with both a CA125 rise and a radiological pelvic mass. Patients were considered ineligible if they had any prior malignancy except basal cell carcinoma or carcinoma *in situ* of the cervix or urinary bladder, prior chemo- or radiotherapy, serious medical or psychiatric illness that might affect treatment, major disturbance of hepatic parameters (alanine aminotransferase or aspartate aminotransferase >3 times the upper limit of normal, total bilirubin >2 times the upper limit of normal), severe renal insufficiency (creatinine clearance <30 mL/min), or abnormal hematological parameters (neutrophils <1.5 × 10^9^/L, platelets <100 × 10^9^/L). Patients with planned interval debulking surgery were also excluded.

### Assessments

A multidimensional pre-inclusion geriatric assessment was performed at baseline. Data concerning the patient's medical charts, nutrition, functionality and an extensive psychocognitive assessment were collected, including comorbities, comedications, body mass index (BMI), serum albumin levels, and functional scores for Activities of Daily Living (ADL), Instrumental ADL (IADL), Geriatric Depression Scale (GDS), Hospital Anxiety and Depression Scale (HADS), and the Mini-Mental Scale (MMS).

Patient outcomes of treatment completion rate (defined as receiving six courses of chemotherapy without premature discontinuation for death, treatment toxicity or tumor progression), survival, serious adverse events, unplanned hospital admissions, and grade ≥3 toxicities were recorded.

The GVS was calculated for each patient as described previously [11]. This score is the addition of the following geriatric vulnerability parameters: ADL score <6, IADL score <25, HADS score >14, albuminemia <35 g/L, and lymphopenia <1 × 10^9^/L.

### Measurement of telomere length

A blood sample was collected at inclusion. DNA extraction was performed within 14 days using the PAXgene Blood DNA System (PreAnalytix GmbH, Hombrechtikon, Switzerland) according to the manufacturer's instructions. DNA integrity was assessed by electrophoresis on 1.0% agarose gels. DNA samples (4μg) were digested overnight with the restriction digest set Hinf1 (33 U) /Rsa1 (33 U) (New Englands Biolabs, France) and resolved using field inversion electrophoresis (FIGE) on FIGE Mapper System (Bio-Rad Life Science, France). Briefly, samples were precipited, resuspended in 12μl of H20 and run on 1% pulse field grade agarose gel (20cm × 13cm) containing 0.5X TBE at room temperature for 13h. The switch time ramp was between 0.1 and 0.5s (linear shape) with forward and reverse voltages of 160 and 100 V, respectively. A combination of two DNA molecular weight size standards was run on each gel : l Mix Marker 19 that spans 48.5 – 1.5kb and MassRuler™ DNA Ladder mix that spans 10 – 0.08kb (Thermo Scientific Molecular Biology Inc., France) and used to establish a standard curve (molecular size as a function of migration distance). Digested DNA were blotted to N+ Hybond membrane (GE Healthcare, France) by capillary transfer using SCC 20× transfer buffer and then UV cross-linked. Hybridization was carried out overnight at 65°C in hybridization buffer (0.5M NaPO4 pH7.2, 7% SDS, 0.1% BSA, 1mM EDTA) containing a digoxigenin (DIG)-labeled probe specific for telomeric repeats (400 bp of repeated 5.-T2AG3-3. motif). Membranes were then washed twice at room temperature in 2X SSC, 0.1% SDS (5min) and twice at 50°C in 0.2X SSC, 0.1% SDS (25min). Chemiluminescence detection was carried out according to TeloTAGGG Telomere Length Assay (Roche Applied Science, France) instructions. Telomeric Restriction Fragments (TRF) chemiluminescence signals were captured using a LAS-3000 Imager (FujiFilm Life Science, France) and images were processed using ImageJ software (http://rsb.info.nih.gov/ij/). The optical densities (OD) versus mean TRF length were calculated according to the formula (∑ ODi/ ∑(ODi/MWi), where ODi is the chemiluminescent signal and MWi is the length of the TRF at position i ([13] and Supplementary Figure 1). Measurements were performed on each sample at least twice in different gels and the mean was used for statistical analyses. Pearson's correlation coefficient for duplicates was 0.74, with an average coefficient of variation for pair sets of 7.3%. The laboratory conducting the TL measurement was blinded to all patient characteristics.

### Statistical analyses

The sample size of 110 patients was calculated on the basis of the primary objective of the main part of the study (to confirm the impact of psychogeriatric covariates on OS) as reported elsewhere [12]. TL was analyzed both as a continuous ordinal variable and a categorical variable. For the former analysis, non-parametric two-sample Wilcoxon rank-sum tests were performed to evaluate the impact of TL on patient outcome. For the latter analysis, TL was transformed into quartiles, categorized into TL groups (shorter vs longer) and introduced as a dichotomous trait into linear regression models and Cox's proportional-hazards regression models. Different cut-offs were used based on the outcome under consideration. Survival curves were estimated using the Kaplan-Meier method and OS models were adjusted for FIGO stage (IV versus III). Odd ratios (ORs) and hazard ratios (HRs), 95% confidence intervals (CIs), and p values (P) were calculated. Analyses were performed using R statistical package (R Foundation for Statistical Computing, Austria) and Splus, version 6.2 (Insightful Corp., WA, USA).

## SUPPLEMENTARY MATERIAL TABLES AND FIGURE


