# Supplementary material for: Telomere length is a prognostic biomarker in elderly advanced ovarian cancer patients: a multicenter GINECO study
Source: Aging (Albany NY). 2015 Dec 3;7(12):1066–74. doi: 10.18632/aging.100840 (PMC4712332; doi:10.18632/aging.100840)
Supplement: Supplementary file 1 [file aging-07-1066-s001.pdf]

SUPPLEMENTARY MATERIAL

Supplementary Table 1. Multivariate models of survival

Risk factors	Model 1	Model 2	Model 3	Model 4
TL < 6·0 kb	HR = 1·57 (95%CI: 0·98-2·51) *P*=0·06	HR = 1·58 (95%CI: 0·99-2·53) *P*=0·06	HR = 1·56 (95%CI: 0·97-2·49) *P*=0·07	HR = 1·57 (95%CI: 0·98-2·53) *P*=0·06
FIGO stage IV	HR = 2·56 (95%CI: 1·54-4·27) *P*<0·01	HR = 2·33 (95%CI: 1·37-3·97) *P*<0·01	HR = 2·41 (95%CI: 1·44-4·05) *P*<0·01	HR = 2·27 (95%CI: 1·33-3·87) *P*<0·01
Age	-	HR = 1·03 (95%CI: 0·98-1·08) *P*=0·21	-	HR = 1·02 (95%CI: 0·98-1·08) *P*=0·34
GVS ≥ 3	-	-	HR = 2·72 (95%CI: 1·64-4·61) *P*<0·01	HR = 2·66 (95%CI: 1·60-4·42) *P*<0·01

Supplementary Table 2. Patients characteristics according TL subgroups

	**N of patients (%)**		**t-Test**
	**ST subgroup (n=33)**	**LT subgroup (n=76)**	***P***
Median age in years (range)	80 (70-93)	78 (70-88)	**0.05**
≥80 years	17 (51·5)	27 (35·5)	0.12
Performance status (ECOG) ≥2	16 (48·5)	35 (46·1)	0.81
Tumor assessment			
FIGO stage IV	9 (27·3)	29 (38·7)	0.26
Complete primary cytoreduction	5 (15·2)	13 (17·1)	0.80
Geriatric assessment			
≥3 comorbidities	10 (30·3)	16 (21.1)	0.30
N comedications			
1-3	8 (24·2)	24 (31·6)	0.44
4-6	13 (39·4)	31 (40·8)	0.89
≥7	12 (36·4)	18 (23·7)	0.18
Functional assessment			
ADL score <6	22 (66·7)	38 (50·0)	0.11
IADL score <25	24 (72·7)	52 (68·4)	0.66
Nutritional assessment			
Albuminemia <35 g/L	20 (60·6)	43 (56·6)	0.70
BMI <21 kg/m²	6 (18·2)	18 (23·7)	0.53
Lymphocyte count <1 G/L	11 (33·3)	16 (21·1)	0.18
Psychocognitive assessment			
MMS score <25	11 (33·3)	21 (27·6)	0.55
HADS score >14	15 (45·5)	25 (32·9)	0.22
GDS score >10	12 (41·4)	24 (33·3)	0.45

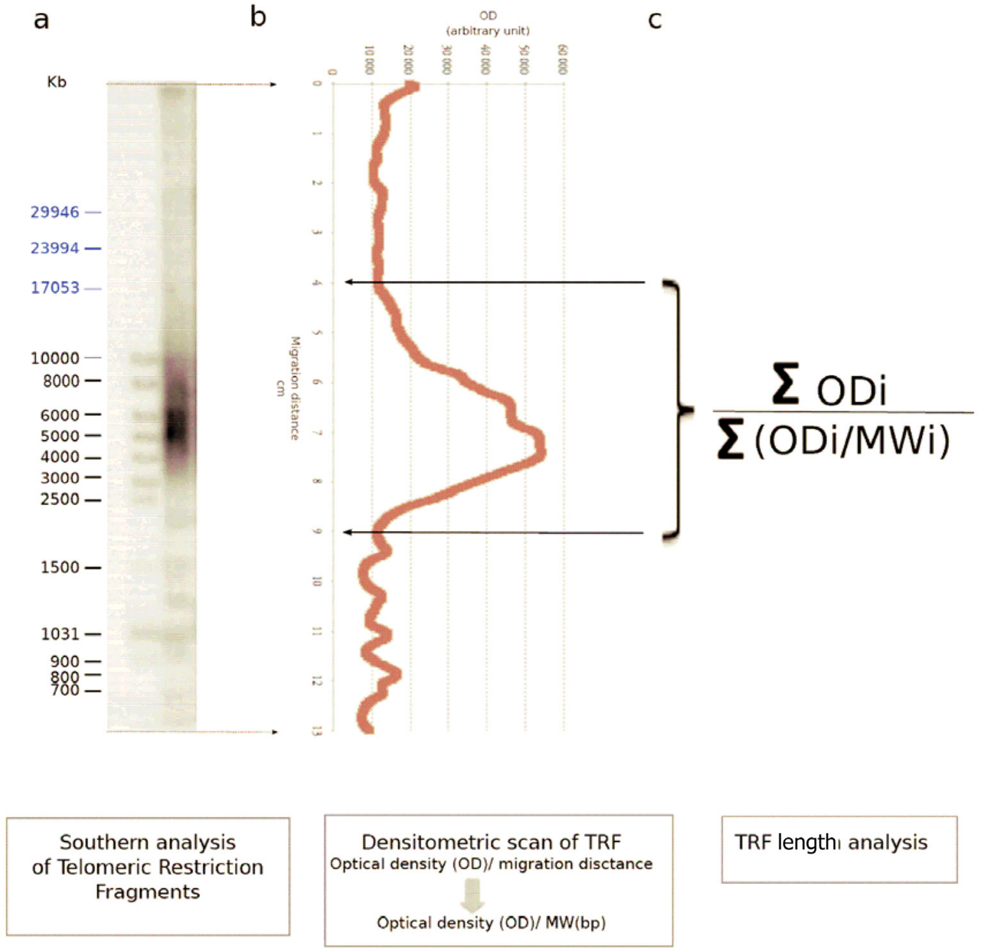

Supplementary figure 1: Distribution of TRF lengths in a representative sample. (a) Southern blot of Telomeric Restriction Fragments using a Dig-labeled probe. Two DNA standards were run on a gel and used to establish a standard curve : *Mix Marker 19* that spans 48.5 – 1.5kb (in blue) and MassRuler™ DNA Ladder mix that spans 10 – 0.08kb. (b) OD versus migration distances derived from (a). Data of OD values versus DNA migration distances were converted to OD versus Molecular Weight using formula relationship between DNA migration distance (y) and MW (x) defined using DNA standard. (c) TRF signals from 3 and 20kb (black arrows) are used for TL measurements. Mean TRF length calculation using the equation: Sum (ODi)/sum(ODi/MWi). The equation was applied to TRF signals falling in the appropriate range determined from the (OD values versus DNA migration distances) plot.
